# Long-term outcomes of trials in the National Institute for Health and Care Excellence depression guideline

**DOI:** 10.1192/bjo.2019.65

**Published:** 2019-09-09

**Authors:** Susan McPherson, Michael P. Hengartner

**Affiliations:** Researcher, School of Health and Social Care, University of Essex, UK; Senior Lecturer and Researcher, School of Applied Psychology, Zurich University of Applied Sciences, Switzerland

**Keywords:** Antidepressants, depressive disorders, individual psychotherapy, outcome studies

## Abstract

The forthcoming National Institute for Health and Care Excellence depression guideline reviews short-term outcomes for long-term depression. We present effect sizes for long-term outcomes in trials that report these data. Psychological therapies become more effective, whereas antidepressants become less effective over the long term. We review other forms of longitudinal research that support these findings.

**Declaration of interest:**

None.

The National Institute for Health and Care Excellence (NICE) have compiled and synthesised a large collection of randomised controlled trials on depression for the forthcoming guideline update.^1^ Trials on long-term forms of depression are grouped into chronic depression and treatment-resistant depression (TRD). Chronic depression trial populations (defined as an episode of at least 2 years) have an actual range of 26–348 months duration.^1^ TRD trial populations have episodes ranging from 0.4 to 92 months; 60% of TRD studies report mean episodes of 2 years or more.^1^ Long-term outcomes in these trials have not been analysed; the review only uses end-of-treatment outcomes, which vary from 1 to 78 weeks, with an average of around 10 weeks. The rationale given is that there are not enough studies with long-term data to analyse.^2^ Stakeholders have argued that long-term outcomes provide the ‘best-possible evidence’ and NICE executives have recently agreed to look at the issue again.^3^ We examine available data to illustrate how NICE could make use of this evidence. Although NICE and other commentators consider TRD and chronic depression to be distinct categories of depression, it has been argued that when subclassified based on limited information available from trials reviewed by NICE, these are not clinically meaningful distinctions.^4^ Specifically, based on the information both present and, critically, absent in the trial papers, there is no reliable basis on which to suppose that these populations are not overlapping. We therefore examine both categories for the purposes of the current commentary.

In chronic depression and TRD categories, 124 trials were included.^1^ Ninety-eight of these included an antidepressant arm (as defined by NICE), 30 included a psychological treatment arm and a proportion of these had both antidepressant and psychological treatment groups. Twenty-two had observation points of 6 months or more. Of these, there were 11 in which the 6-month observation was at end of treatment, rather than ‘follow-up’. Without a period between end of treatment and follow-up, it is not possible to form a view on whether the effects of the treatment last beyond end of treatment. Table 1 collates information for the remaining 11 trials: we report the amount of change on continuous symptom scales at end of treatment and follow-up for each of the trials with long-term follow-up data via Cohen's *d* effect sizes. Trials are ordered by group comparison effect size at follow-up.

**Table 1:** Effect sizes at end of treatment and follow-up

Study^24–34^	Classification in NICE guideline	Current episode duration (mean months, s.d.)	*N*^a^	Measure for effect size^b^	Treatment duration (weeks)	Follow-up (weeks from baseline)	Effect size (end of treatment)		Effect size (follow-up)	
							Baseline to end^c^ (95% CI)	Group comparison^d^	Baseline to follow-up^c^ (95% CI)	Group comparison^c^
Paykel 1999	TRD	13.8 (median)	158		20	68	Cognitive therapy plus ADM: NDA ADM: NDA	NDA	Cognitive therapy plus ADM: NDA ADM: NDA	NDA
Valenstein 2015	TRD	Not reported	334	BDI-II	24	52	Peer support: 0.59 (0.33–0.85) TAU: 0.62 (0.43–0.80)	−0.03	Peer support: 0.72 (0.46–0.98) TAU: 0.71 (0.52–0.90)	0.01
Hellerstein 2001^e^	Chronic depression	Not reported	32	HRSD	16	36	CIGP + ADM: 0.19 (−0.45 to 0.83) ADM: 0.13 (−0.52 to 0.79)	0.06	CIGP + ADM: −0.07 (−0.71 to 0.56) ADM: −0.02 (−0.68 to 0.63)	0.05
Browne 2002^e^	Chronic depression	Not reported	586	MADRS	26	104	IPT: 1.28 (1.06–1.49) IPT + ADM: 1.28 (1.07–1.49) ADM: 0.92 (0.70–1.14)	IPT versus ADM: 0.36	NDA	IPT versus ADM: 0.27
Wiles 2013^e^	TRD	Not reported	408	BDI-II	27	52	CBT + ADM: 1.04 (0.84–1.24) ADM: 0.61 (0.42–0.80)	0.43	CBT + ADM: 1.21 (1.00–1.42) ADM: 0.85 (0.65–1.05)	0.36
Schramm 2011^e^	Chronic depression	243.6 (135.6)	29	BDI-II	16	52	CBASP: 1.64 (0.78–2.49) IPT: 0.61 (−0.13 to 1.34)	0.87	CBASP: 1.17 (0.37–1.97) IPT: 0.83 (−0.08 to 1.58)	0.43
Schramm 2015^e^	Chronic depression	Not reported	59	MADRS	8	28	CBASP: 0.42 ADM: 0.63	−0.21	CBASP: 0.98 ADM: 0.50	0.48
Fonagy 2015^e^	TRD	45.0 (36.4)	129	HRSD	78	182	LTPP: 0.60 (0.25–0.95) TAU: 0.40 (0.05–0.76)	0.20	LTPP: 0.65 (0.30–1.00) TAU: 0.02 (−0.33 to 0.37)	0.63
Chiesa 2015^e^	TRD	25.5 (47.9)	43	HRSD	8	26	GMBCT + ADM: 1.12 (0.50–1.74) Psychoeducation plus ADM: 0.29 (0.33–0.92)	0.83	GMBCT + ADM: 1.54 (0.88–2.20) Psychoeducation plus ADM: 0.39 (0.23–1.02)	1.15
de Mello 2001	Chronic depression	Not reported	23	HRSD	12	48	IPT + ADM: 2.84 (1.76–3.19) ADM: 2.65 (1.69–3.60)	0.19	IPT + ADM: 3.67 (2.43–4.91) ADM: 2.46 (1.51–3.40)	1.21
Schramm 2008	Chronic depression	Not reported	37	HRSD	5	52	IPT + ADM: 2.56 (1.80–3.32) ADM: 1.44 (0.76–2.12)	1.12	IPT + ADM: 4.19 (3.12–5.28) ADM: 1.52 (0.81–2.22)	2.67

NICE, National Institute for Health and Care Excellence; TRD, treatment-resistant depression; ADM, anti-depressant medication; NDA, no data available (missing means and/or s.d. to allow calculation); BDI-II, Beck Depression Inventory-II; TAU, treatment as usual; HRSD, Hamilton Rating Scale for Depression; CIGP, cognitive interpersonal group psychotherapy; MADRS, Montgomery–Asberg Depression Rating Scale; IPT, interpersonal therapy; CBT, cognitive–behavioural therapy; CBASP, cognitive–behavioural analysis system of psychotherapy; LTPP, long-term psychodynamic psychotherapy; GMBCT, group mindfulness base cognitive therapy. For detailed information about measures and references, see the full guideline updated version.^1^

a. Number at last time point.

b. Measures selected based on what data were available at the follow-up point.

c. Effect sizes reported are Cohen's *d* calculated from raw mean and s.d. weighted by sample size (except Schramm 2015 where the author provided effect size for end follow-up are used and hence no confidence interval is reported).

d. Group comparison effect size calculated as effect size 1 minus effect size 2 (except Browne 2002 baseline follow-up, which is calculated from author reported change scores).

e. Intention-to-treat analysis (ITT) or modified ITT (participants who attended a minimum of one session).

Time between end of treatment and follow-up ranged from 18 to 104 weeks, which limits comparability. In addition, in the smaller studies, the confidence intervals were quite wide, which limits the degree of certainty in the findings. However, examining pre–post effect sizes by intervention arm indicates that most psychological treatments for both TRD and chronic depression appear to become more effective at follow-up, with the exception of cognitive interpersonal group psychotherapy, which appears to become less effective, and cognitive–behavioural analysis system of psychotherapy, which has mixed results. The latter two psychological treatments were examined in populations classified by NICE as chronic depression. Examining group comparison effect sizes at follow-up suggests that antidepressants are consistently less effective than either psychological treatment alone or in combination with antidepressants over the long term.

There are limitations to comparing antidepressant and psychological treatment trials, which apply to all the trials reviewed, including inflation of outcomes from different comparator arms (only two of the trials directly compared antidepressants alone with psychological treatment alone); effects of completer versus intention-to-treat (ITT) analyses (half of the trials here used ITT or modified ITT requiring a minimum of one session attendance); inflation of effects from non-blinding in psychological treatments; and differential effects of under-reporting harms, side-effects and tolerability in psychological treatment versus antidepressant trials. Therefore, the findings here are not presented as a standalone meta-analysis and should be interpreted with caution. NICE guidelines provide grading of recommendations assessment, development and evaluation (GRADE) of all trials to take these sorts of bias into account when translating findings into recommendations. Although the draft depression guideline has produced preliminary GRADE assessments, these cannot yet be directly applied to the current analysis because they remain under review and stakeholders have raised significant concerns about the way in which GRADE was applied.^2^ Of particular relevance to the current analysis is the concern that trials were downrated on quality if the 95% confidence interval crossed both line of no effect and threshold for clinically important benefit at the end of treatment, irrespective of whether this had ceased to be the case at a follow-up data point, which stakeholders argue prejudices trials where the effect emerges at follow-up. Also relevant is the concern that trials were downrated on quality where participants were not blinded; stakeholders argue this systematically prejudices psychological treatment trials in which concealment of the treatment arm is impossible.

Although it remains necessary to be cautious about the tentative findings presented, it is also possible to consider other forms of longitudinal data that appear to support the findings. Discontinuation trials, for example, were designed to test the efficacy of staying on antidepressants long-term compared with stopping and switching to placebo. About a third of patients remaining on antidepressants relapse within 6 months of maintenance therapy.^5^ Remaining on antidepressants for more than 18 months appears to prevent immediate relapse but, when antidepressants are then discontinued, recurrence is very common. Indeed, recurrence is more likely the longer the antidepressant has been taken and is more likely for patients using antidepressants than for trial participants who remitted while on placebo.^6,7^ These findings could be a result of ‘oppositional tolerance’ or neurobiological adaptation to antidepressants worsening the long-term course of depression.^8^ The picture is further complicated, however, because any trial examining ‘relapse’ as an outcome of stopping antidepressants could be conflating relapse with withdrawal symptoms.^9^

Various naturalistic cohort studies show that long-term antidepressant use has worse outcomes than short-term antidepressant use or non-pharmacological treatments.^10,11^ Moreover, it appears to be difficult to stop antidepressants after extended use;^12^ some patients using antidepressants may develop dependency^13^ and patients using antidepressants long term report feeling addicted.^14^ Safety reviews find that long-term antidepressant use is associated with serious health risks, including obesity, hepatotoxicity and cardiovascular events.^8^

Meta-analyses of direct comparisons in randomised controlled trials for (recurrent) major depressive disorder (MDD) consistently show that, in protecting against relapse in the long term, psychological treatment is superior to maintenance antidepressant use.^15^ In combination for up to 6 months, antidepressants plus psychological treatment appears to be more effective for MDD than psychological treatment or antidepressants alone; however, long-term follow-ups reveal that beyond 6 months, there is no advantage of combination treatment over psychological treatment alone.^16^ Two reviews also found that (mindfulness-based) cognitive–behavioural therapy following antidepressant discontinuation was more effective than antidepressant continuation.^17,18^

Treatment comparison studies suggest that long-term psychological treatment irrespective of modality generates large effect sizes over the long term for MDD and chronic depression. For example, comparisons of cognitive–behavioural therapy and psychoanalytic psychotherapy find roughly equal but strong effect sizes after 1 year^19^ and 3 years.^20^ Similarly, a systematic review found that long-term relapse rates in depression over 2 years are lower for psychological treatments than other interventions.^21^ In contrast, less than 10% of patients treated with antidepressants achieve sustained remission over 1 year in representative real-world effectiveness trials.^22^ A systematic review of naturalistic studies of antidepressant use of at least 10 years’ duration revealed that only about a quarter of people improved or remitted, and another quarter were classified as severely impaired and ill-functioning.^23^

## Conclusion

If it is possible to agree that good-quality, long-term trial data is the best possible evidence for long-term conditions, then the paucity and variable quality of this data should not be a reason to exclude it in analyses used to inform depression guideline recommendations. Tentative analyses of long-term outcome data reveal an important clinical picture, which is supported by evidence from other forms of longitudinal research. Although the evidence remains to be appropriately evaluated with GRADE methodology, it is nevertheless important to consider it in the guideline because of the effect NICE guidelines have globally on patient care as well as ongoing and future research priorities.

## Data Availability

Sources of information used were NICE guideline drafts, documents and appendices all available on the NICE website.^1^ Specifically, Appendix J5 and J6 were used, which detail all trials reviewed in the TRD and chronic depression categories. See https://www.nice.org.uk/guidance/gid-cgwave0725/documents/addendum-appendix-9 and https://www.nice.org.uk/guidance/gid-cgwave0725/documents/addendum-appendix-10. Note that Town 2017 was included in the ‘Full guideline updated’ but omitted from Appendix J5.
